# Functional and Structural Details about the Fabella: What the Important Stabilizer Looks Like in the Central European Population

**DOI:** 10.1155/2015/343728

**Published:** 2015-08-27

**Authors:** Nicole Helene Hauser, Sebastian Hoechel, Mireille Toranelli, Joerg Klaws, Magdalena Müller-Gerbl

**Affiliations:** Department of Biomedicine, Musculoskeletal Research, University of Basel, Pestalozzistrasse 20, 4056 Basel, Switzerland

## Abstract

The posterolateral corner of the knee accommodating the fabella complex is of importance in orthopaedic surgery. Unfortunately, there is a lack of data in literature for clinical routine. Therefore, we investigated the fabella's characteristics, biomechanical nature, and present histologic details. Of special interest were the fabella's occurrence and position, calcium concentration as long-term load intake indicator, and the histology. Within our analysis, fabellae were found in 30.0% of all datasets, located on the upper part of the posterolateral femoral condyle. The region of fabella contact on this condyle showed a significantly lower calcium concentration than its surroundings. Histologically, the fabella showed no articular cartilage but a clearly distinguishable fabellofibular ligament that consisted of two bundles: one, as already described in literature inserted at the fibular tip, and another part newly described on the top of the lateral meniscus. In its role of stabilizing the soft tissue structures of the posterolateral knee, the fabella seems to serve as suspension for the ligaments evolving from its base. Even though a joint formation of any kind is unlikely, the presence of a fabella needs to be kept in mind during knee examination and any surgical procedures.

## 1. Introduction

The occurrences of patients with knee injuries are constantly rising. Though injuries of the medial compartment are more frequent, the consequence of a traumatic distress on the lateral side is more disabling, since the lateral compartment is subjected to greater force during gait [1, 2]. In addition, possible posterolateral pathology may remain unrecognized if injuries of the cruciate ligaments mask secondary findings due to their extensive symptoms [3, 4]. In its stabilizing function, the posterolateral corner with its complex arrangement of muscles, tendons, and ligaments is of crucial importance for the physiological function of the knee. It is suggested that untreated damage and therefore insufficient support of the posterior knee not only prolong the healing process, but also cause postsurgical failure after cruciate ligament reconstruction [5, 6]. Since the injury occurs following direct varus force in external rotation of the tibia as well as sudden hyperextension of the knee, various case reports describe traumata after car accidents with frontal impact not only to the ligamentous structures, but also to the occasional fabella present [7–11]. To understand the possible injuries and repair mechanisms of the posterolateral corner, the arrangement and variation of anatomical structures must be considered. A literature review of the fabella is confusing, since information about the fabella complex is obtained by many different research methods. Unfortunately, the derived results are still compared to each other which results in a mix of data which is difficult to handle. Despite the fact that information about the central European population is negligible [12], various numbers of Chinese and Japanese studies report the occurrences of the fabella positioned in the lateral head of the gastrocnemius muscle and the possible structure of the fabellofibular ligament (FFL) [13, 14].

Tabira et al. report in 2013 the prevalence of the fabella to be 68.6 percent (%) per knee in the elderly Japanese population. Included were bony and cartilaginous findings evaluated by inspection and palpation within the lateral head of the gastrocnemius muscle [15]. On the contrary, Kawashima et al. report their results of 66% bony and cartilaginous fabellae present in a similar Japanese study per gastrocnemius head and not per knee [14]. In addition, some studies conduct their focus on clinical radiographs which mainly take into consideration the prevalence of osseous fabellae [16]. The main confusion arises, when these findings are compared with each other and the methods of examination are not clearly stated [17].

Despite the differences in these data, the description of the endochondral ossification and the occurrence and structure of the FFL is consistent. Running from the base of the fabella to the styloid process of the fibular head, it serves as a static stabilizer of the knee, which tenses in full extension [18]. It can be found in up to 80% of humans with a fabella present [16, 19, 20].

For the orthopaedic surgeon, this information provides ideas about the posterolateral knee complex and helps to tackle arising symptoms like pain and swelling in this area, especially if the interaction of the fabella and the posterolateral femoral condyle (PLFC) is taken into account. Many researchers favour the idea of a fabellofemoral joint with typically associated joint diseases like chondromalacia and osteoarthritis, which can be found in many case reports [7, 11, 21]. In progressive stages, the cartilage of the fabella is described as softened and fibrillated or even completely absent. In this case, the bare subchondral bone plate of the fabella comes into contact with the femoral condyle and leads to increasing posterolateral pain [22]. The gross anatomy of the proclaimed joint in a healthy state and the formation of the interacting surfaces are difficult to retrieve from literature. The description of the joint cavity was done by gelatine-injection, which was not characterized any further and documented in black and white pictures in which the markings hide the important areas. What can clearly be identified is the distinctive impression on the PLFC caused by the fabella [14, 17]. Histologic images of the fabella and the surrounding formation lack representation in literature. Most of the presentations are very small and reveal only fractions of the posterolateral aspect of the knee. In addition, the images available are mainly printed in black and white and are not connected to an overview presentation [17, 20, 23, 24]. In these studies, the cartilage formation and the interaction with the femoral condyle are not sufficiently described and do not provide conclusive information about the anatomical arrangement in order to evaluate and understand patient cases better.

Our goal was to (1) determine the incidence and position of the fabella in a central European population and to better estimate clinical appearances. (2) Furthermore, we describe the biomechanical impact of the little sesamoid bone on its interaction with the femur in order to determine a possible pressure distribution. (3) Histologic demonstration will define the anatomical structures with special attention to the ligamentous tissue and clarify the formation of the existing cartilage in order to determine any possible joint formation. The arrangement of the structures of the posterolateral knee will be shown in its entirety including all bony surroundings. A stained overview presentation is not yet available in literature, since deficient bone decalcification has hampered the production of cuttings that include materials with different rigidity.

## 2. Materials and Methods

### 2.1. Sample Collection

Four hundred knees of 200 Europeans were included (cadaveric study group-corpses donated to science and research, 99 men, 101 women; conventional datasets generated by computed tomography (CT), extended knee position). The data was acquired during investigations at the Institute of Anatomy, University of Basel. The sample age ranged from 20 to 104 years (mean: 75.8, SD: 19.43). Histologic procedures were carried out selectively on five of the most prominent and representative bony fabella samples.

### 2.2. Descriptive Quantitative Analysis

**Figure 1:**
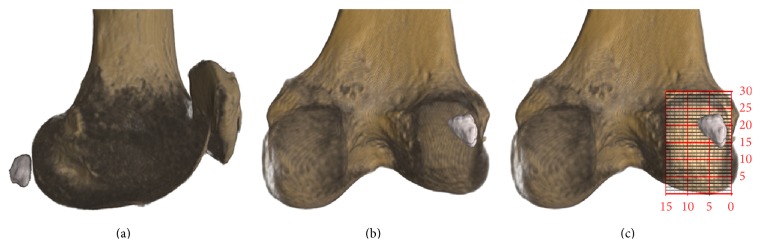
3D reconstructions for descriptive quantitative analysis. (a) 3D reconstruction of the distal femur, patella, and fabella in sagittal view. (b) 3D reconstruction of (a) positioned in posterior view. (c) Applied coordinate-grid system on the posterolateral femoral condyle for determination of fabellar position.

We evaluated CT-studies (SOMATOM 16, Siemens, Erlangen, Germany, 120-kilovolt, 180-milliampere-second, axial slices) with a slice thickness of 0.6 millimetres (mm) (only the bony fabella could be registered with the CT-method). Three-dimensional (3D) reconstructions (ANALYZE 11.0, Biomedical Imaging Resource, Mayo Foundation, Rochester, USA; VGStudio Max 2.2, Heidelberg, Germany) were orientated in coronal posterior view for determination of the location of the fabella. To gain comparable data, a size-independent measurement grid system was applied on the PLFC to allocate the corresponding coordinates to the centre of the fabella (Figures 1(a)–1(c)). The determined location coordinates of every fabella were superimposed on one reconstructed 3D-sample of the PLFC with respect to the anatomical orientation for final evaluation.

To determine the size of the fabella, the largest diameter (*x*) was measured (SOMATOM 16, Siemens, Erlangen, Germany) in coronal posterior view, regardless of the anatomical knee-axes. For the second dimension, the largest diameter of the corresponding perpendicular orientation (*y*) was used.

### 2.3. Joint Impact Analysis

The method of CT-osteoabsorptiometry (CT-OAM) for assessment of the density distribution was used on the same 3D reconstructions of the conventional datasets from the descriptive analysis described above. The 3D reconstructions of the knee were divided into datasets of the fabella and the femoral component. The subchondral bone plate (SBP) of the PLFC and of the fabella as the region of interest was arranged in a way to be in coronal frontal view. Using a “maximum intensity projection” the software (ANALYZE 11.0, Biomedical Imaging Resource, Mayo Foundation, Rochester, USA) projected the most dense voxels onto the surface and assigned them colours, where the highest density values (>1200 Hounsfield units) were represented in black and lower values in red, yellow, green, and blue (in descending order) [25, 26]. In accordance with the density values displayed, phantom measurements led to the calculation of the corresponding mineral content as an indication of the long-term load intake [27].

**Figure 2:**
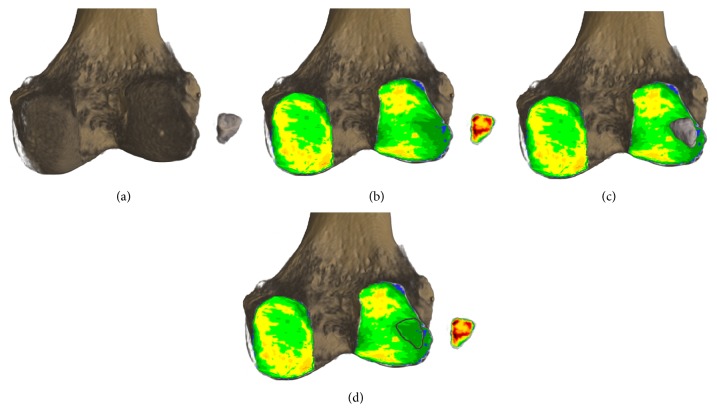
Method of CT-osteoabsorptiometry on 3D reconstructions to determine density distribution within the subchondral bone plate. (a) Distal femur in posterior view with fabella arranged in anterior view (subchondral bone plate of articular surface shown). (b) Subchondral bone plates shown with colour-coded density distribution (black, red, yellow, green, and blue = Hounsfield units in descending order in 200 unit steps). (c) Fabella in original position on density distribution pattern. (d) Density distribution with marked region of fabella contact on the posterolateral femoral condyle.

For statistical analysis, the mean concentration of calcium hydroxyapatite/Ca_5_(PO_4_)_3_(OH) (subsequently abbreviated as CAHA) within the SBP of the fabella and the corresponding area of contact on the PLFC was evaluated. In addition, the mineral content of the SBP of both posterior femoral condyles was measured for reference (Figures 2(a)–2(d)).

### 2.4. Histologic Imaging

For sectioning and staining, the five largest fabella samples were dissected and removed with the attached soft tissue structures as well as the corresponding PLFC.

The dissected tissue was treated to dehydration in ethanol starting with 40% increasing to 100% over a time period of 25 days. Afterwards, the initial defatting process was increased using isopropyl alcohol and chloroform. After completion, the next step included methyl methacrylate (MMA) infiltration for 3 days at a storage temperature of 4° Celsius. Following this step of infiltration and mixing of chemicals, the resulting solution was exchanged for pure MMA again for the final embedding at 4° Celsius. The time of polymerization was in accordance with the size of the sample and lasted approximately one month. For all further steps, the hardened MMA blocks embedding the bone samples were used.

The sectioning in sagittal anatomical orientation was performed using a diamond wheel saw with 400-micrometre (*μ*m) saw band thickness. The resulting slices (thickness: 600 *μ*m) were fixed on white, light-transmissive object holders for further processing. To accomplish optimal staining conditions, the slides were ground down to 200 *μ*m and polished. Staining methods obtained the following.

- Toluidine blue epoxy staining of 3 *μ*m [28], for basophil structures to acquire different shades of blue where calcified cartilage shows the darkest shade,
- Trichrome Masson-Goldner surface staining of 3 *μ*m [29], where mineralized bone and collagen are stained green, calcified cartilage is stained light green, and muscle tissue and cytoplasm are stained in different shades of red. The resulting histologic slices were documented for inspection (20.5 : 1 Zoom and FusionOptics Technology Leica M205 C; Canon EOS 40D).

### 2.5. Statistical Analysis

Continuous variables were expressed with mean, standard deviation, and minimum-maximum values where categorical variables were reported as frequency and related percentage. Independent samples* t*-test was performed between group comparisons. Linear regression analysis was performed for modelling the relationship between PLFC, FAS, and ROFC. All age group data were tested for normalcy and homogeneity using Kolmogorov-Smirnov tests. For the gender distribution analysis, the unpaired two-sample* t*-test was used.

All statistical analyses were done using RStudio (RStudio: integrated development environment for R, Version 0.96.122, Boston, MA, USA). The significance level for all statistical tests was set a priori to <0.001.

## 3. Results

### 3.1. Descriptive Quantitative Analysis

**Figure 3:**
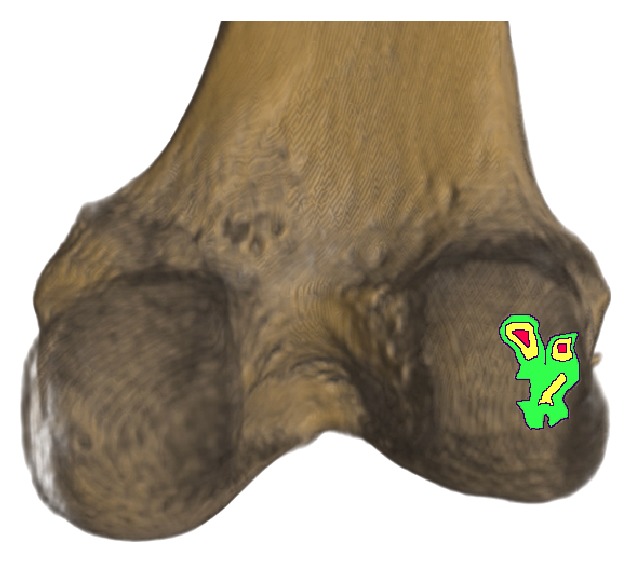
Results of descriptive quantitative analysis. Colour-coded fabella positions on posterolateral femoral condyle (red, yellow, and green; in descending order).

30.0% of all CT-studies (each CT-study of one human included the left and right knee) presented with 105 bony fabellae overall where the bilateral to unilateral occurrence ratio was 3 : 1 (bilateral: 45; unilateral: 15). The relative occurrence showed no significant (*P* > 0.001) difference between the assigned age groups, where fabellae were present (mean occurrence: 23.63%; min: 12.5%; max: 31.54%; SD: 6.71%). The data did not reveal any difference in gender distribution (fabellae in male versus fabellae in female: 1 : 1; *P* = 0.453) (Table 1). The fabella was positioned invariably over the PLFC and in close relation to its lateral border (Figure 3). The measured sizes of the analysed fabellae ranged from (*x*) 4.84 mm, (*y*) 3.63 mm to (*x*) 13.12 mm, (*y*) 11.71 mm.

### 3.2. Joint Impact Analysis

The CAHA concentration of the posteromedial femoral condyle (mean: 461.14 mg/mL; min: 282.66 mg/mL; max: 656.63 mg/mL; SD: 112.93 mg/mL) was significantly (*P* < 0.001) higher compared to the PLFC (mean: 402.59 mg/mL; min: 260.16 mg/mL; max: 577.28 mg/mL; SD: 92.82 mg/mL), hosting the fabella. The measured concentration of the region of fabella contact (ROFC) on the PLFC was significantly (*P* < 0.001) lower (mean: 336.77 mg/mL; min: 198.23 mg/mL; max: 521.98 mg/mL; SD: 91.20 mg/mL) than the mean value measured over the whole PLFC itself. The mineral content of the ROFC, in comparison to the mineral content on the whole PLFC, showed a mean difference of −16.59% (min: −1.77%; max: −33.53%).

**Figure 4:**
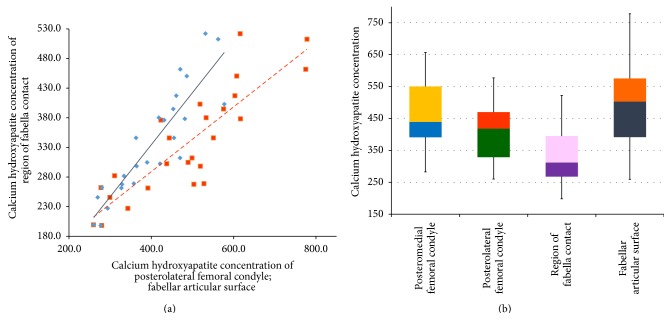
Results of calcium hydroxyapatite analysis of the subchondral bone plates. (a) Level of calcium hydroxyapatite (mg/mL) of the subchondral bone plates of the articular surfaces of interest. (b) Dependency of the evaluated data of the region of fabella contact on the posterolateral femoral condyle (*y*; mg/mL) to the concentration on the mean posterolateral femoral condyle and the fabellar articular surface (*x*; mg/mL).

The fabellar articular surface (FAS) had the highest CAHA concentration (mean: 487.09 mg/mL; min: 259.29 mg/mL; max: 778.15 mg/mL; SD: 142.99 mg/mL) which was on the same level as seen in other joints of the human body (Figure 4(a)).

The linear regression (of CAHA concentration) of the ROFC is dependent on the PLFC and can be interpreted as

(i) ROFC = 0.878 × PLFC − 16.843 (*R* ^2^ = 0.80; *P* < 0.001).

As for the CAHA concentration of the ROFC which is dependent on the FAS:

(i) ROFC = 0.546 × FAS + 70.79 (*R* ^2^ = 0.73; *P* < 0.001) (Figure 4(b)).

### 3.3. Histologic Imaging

**Figure 5:**
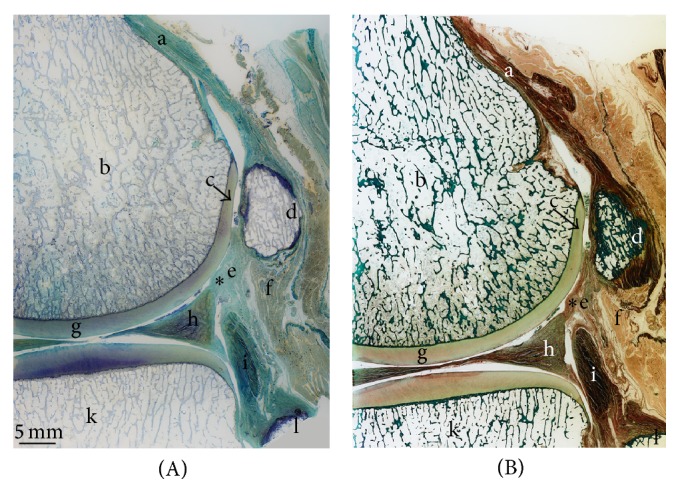
Histologic imaging of posterolateral corner of the knee (sagittal section). (A) Toluidine blue staining. (B) Trichrome Masson-Goldner staining. On both (A) and (B): a: gastrocnemius tendon; b: posterolateral femoral condyle; c: femur condyle impression; d: bony fabella; e: collagen fibres of fabellofibular ligament; f: muscle cells of lateral gastrocnemius head; g: femoral articular cartilage; h: lateral meniscus; i: popliteus muscle; k: lateral condyle of tibial plateau; l: fibular head; ∗second bundle of the FFL.

Represented on the sagittal sections of the posterolateral corner of the knee, the macroscopic images involve all corresponding bones (Figures 5(A) and 5(B)). The fabella was located within the lateral gastrocnemius tendon. The collagen fibres can be found along the anterior and posterior sides of the fabella, joining again to form the muscle-tendon junction at its base. The concave imprint on the articular cartilage of the femur induced by the fabella is formed in the topmost region of the articular cartilage of the PLFC (Figure 5; arrow). The FFL originating from the base of the fabella crosses over the popliteal tendon and is inserted at the styloid process at the tip of the fibula. A second bundle of the FFL can be identified which separates from the main bundle and is inserted at the topmost rim of the lateral meniscus.

**Figure 6:**
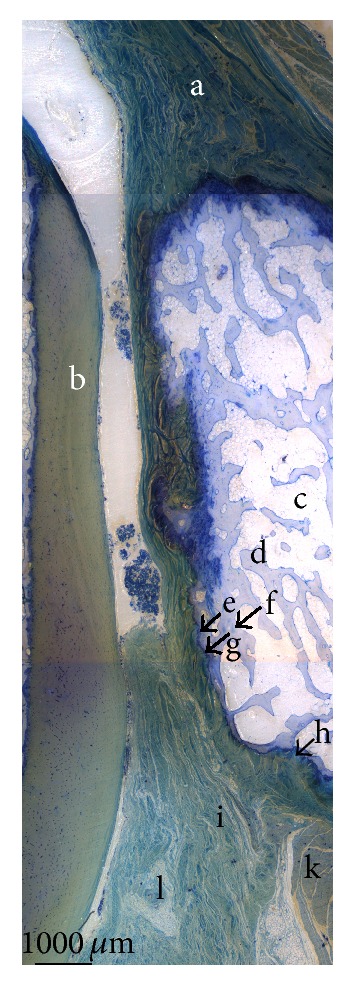
Fusion of magnified histological sections (1000 *µ*m; Toluidine blue staining). a: gastrocnemius tendon; b: femoral articular cartilage; c: medullary cavity of fabella; d: trabecular bone; e: mineralized fibrocartilage; f: osteocytes; g: tidemark; h: unmineralized fibrocartilage; i: collagen fibres of the fabellofibular ligament; k: muscle cells with nuclei; l: fibrocyte.

The magnified image (Figure 6) demonstrated different zones of the femoral articular cartilage with its subsequent tidemark, calcified cartilage, and the SBP. The corresponding surface of the fabella is composed of collagen fibres originating from the gastrocnemius tendon. Just below these longitudinal structures, an unmineralized fibrocartilage followed by tidemark and mineralized fibrocartilage is distinguishable. The medullary cavity within the fabella consists of a clearly defined trabecular network including osteocytes. The accumulation of unmineralized and mineralized fibrocartilage in the middle part of the anterior side of the fabella demonstrates the beginning of pathologic thickening.

## 4. Discussion

The frequency of occurrence of the fabella is discussed in different ways in literature. While Kawashima et al. [14] report cartilage and bony fabellae to be present in 66% of 150 gastrocnemius heads, the comparing paper of Tabira et al. [15] calculated their 68.6% per knee. Another paper, referring to Kawashima et al., quoted them with 92%, a number which does not appear in the paper at all [17]. The confusion arises from different mathematic procedures, calculated either per person, per knee, or per gastrocnemius head. The interpretation of the described differences is mainly based on the state and formation of the fabella, classified as either bony and cartilaginous or soft and hard [14–17, 20, 24]. CT-data will only show bony samples, whereas dissection may derive both and enlarge the number of findings [4]. The so-called commonly known fact that a fabella ossifies at 3 years of age confronts the idea of an induced ossification with aging [6, 14]. Following the data of Minowa et al. who found bony fabellae in fetuses already, one has to rethink about the ossification timeframe mentioned above. To provide reliable data of the central European population that adds to the present state in literature and describes details of the fabella for clinicians, we limited our study to CT-recognizable, bony appearances as they will be the ones discovered in clinical routine. Our findings are in accordance with the commonly understood 30% of fabellae present [16]. Within our study, the distribution of occurrence proved to be quite consistent regarding the patients' age. It is for sure that intrinsic genetic factors as well as extrinsic epigenetic stimuli trigger the ossification of this sesamoid bone. One interpretation that it is due to the aging process is not supported by our data. Reasons for the absence of fabellae in the age groups 20–29 and 100–109 will presumably be the limited number of CT-datasets.

All evaluated bony fabella samples were situated within the tendon of the lateral gastrocnemius muscle and in close relation to the lateral border of the PLFC. In contrast to the literature, we observed the majority located within the superior lateral area. Despite the previous reported main location of the fabella the inferior lateral area of the PLFC [14, 15], we only found about 30% of all fabellae located there.

**Figure 7:**
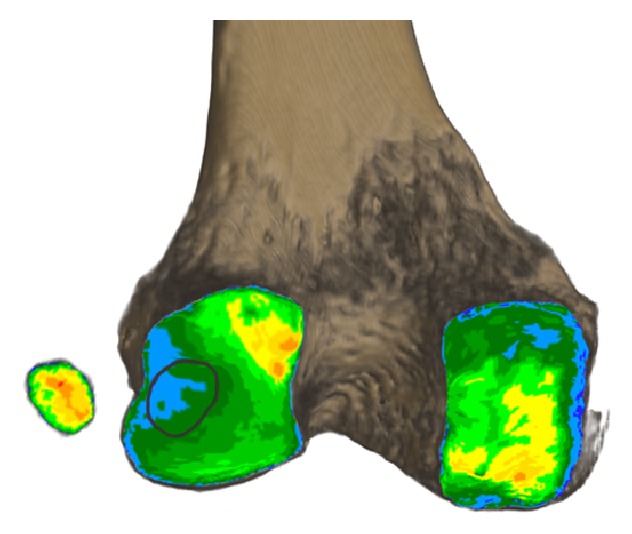
Density distribution of subchondral bone plates of the fabella and femur. Distribution pattern with marked region of fabella contact on the posterolateral femoral condyle.

The data of the long-term loading history evaluated with the method of CT-OAM revealed surprising results. Since the mineralization distribution of the SBP changes in adaptation to the long-term load intake of a joint by CAHA integration and degradation and correlates directly with its mechanical strength, the distribution of the mineral content within a joint surface can be regarded as a reflection of the load intake over time and represents the loading history [25, 30–32]. Due to the fact that the interacting parts of the femoral condyle and the fabella are described as a joint, we expected joint-loading to be represented in the density distribution pattern [14]. The PLFC, however, showed to be less mineralized in the ROFC than the rest of the evaluated area. The contour of the fabella itself is even recognizable as it is coded in a different colour resembling a lower mineral content (Figures 7 and 2(d)). A joint formation between the corresponding bones does not seem to exist here. In addition, the histologic pictures support these findings with the absence of articular cartilage and a cover of collagen fibres on the fabella. The SBP of the fabella meanwhile shows a mineral content that is on a comparable level to other joints [27]. The fact of the lower load uptake of the ROFC leads us to take the knee-biomechanics into account for explanation. Within its fabella complex, the sesamoid bone serves as the combined origin of the oblique popliteal ligament, arcuate ligament, and the FFL as well as the plantaris muscle. All these structures fix the sesamoid bone in its position within the gastrocnemius head. Due to the rollback of the lateral femoral condyle during knee flexion, the PLFC moves on the tibial plateau over a greater distance than the medial one. From 120° of flexion, the lateral femoral condyle moves 23 mm in anterior direction until −5° of extension. On the medial side, the contact point only moves 3 mm [19]. This kinematic condition produces more tensile stress on the lateral head than on the medial one which might serve as an extrinsic epigenetic stimulus to trigger the calcification but surely separates the PLFC from the fabella and reduces the impact as shown.

The histologic images in their representation of the posterolateral knee complex add to the current available data in literature, since they include a stained overview, as well as detailed information about the structural composition of the bony components as well as soft tissue. In addition to the already described imprint of the fabella onto the femoral articular cartilage, the tendon of the gastrocnemius muscle can clearly be identified surrounding the fabella and embedding it. Articular cartilage on the fabella is missing. In addition to the already in literature described FFL (originating from the base of the fabella and being inserted at the styloid process of the fibula), a second bundle of collagen fibres is present. Also originating at the base, it separates from the main FFL and is inserted at the topmost corner of the lateral meniscus (Figures 5(A) and 5(B)) [18]. A posterior fixation of the lateral meniscus is therefore possible through this ligamentous bundle. This constellation, however, forms a capsule-like surrounding of the fabella which might be the explanation for the observed articular cavity described by Kawashima et al. [14].

Possible limitations to this study are seen in the pathologic alterations of the histologic samples. An osteoarthritic process can be found in the middle part of the fabella. Since the continuity of the collagen fibres is clearly visible in its full extent, we nevertheless regard this information to be representative.

## 5. Concluding Remarks

A fabella is present within the posterolateral knee complex in 30.0% of the European population and needs to be distinguished from any fracture parts suspected within this area. In its function of supporting the soft tissue structures, it imprints on the articular cartilage of the PLFC in close relation to its lateral border where it is constantly found in CT-datasets if present. Although this close relation created an imprint on the femoral articular cartilage that proves interaction between the fabella and the PLFC, the SBP of the femoral part does not reveal any signs of long-term loading from the fabella in this area. The fabella itself shows no sign of articular cartilage. Instead, it is isolated from the femur, just being surrounded by fixating collagen fibres originating from the lateral head of the gastrocnemius muscle. In its role of stabilizing soft tissue structures, it seems to serve as suspension for the ligament evolving from its base. Despite previous descriptions of this FFL running distally and being inserted at the styloid process, we clearly identified a second bundle inserted into the top rim of the lateral meniscus, which we assume provides mechanical support and a possible back-tracking of the lateral meniscus during its sliding movement on the lateral tibial condyle. Certainly, within the complex field of traumatic knee injuries, a distortion with damage to the lateral meniscus is bound to damage this ligamentous structure as well. Next to the described fabella, the FFL with its second, meniscal attached bundle needs to be kept in mind during knee examination.

## Figures and Tables

**Table 1:** Fabella occurrence in accordance with selected age groups.

Distribution of fabella occurrence								
Age group	*n* ^*^	Male	Female	Number of knees	Fabellae present	Bilat./Unilat. per *N* ^**^	Percentage per age group (%)	Relative distribution
20–29	5	3	2	10	0	0	0.00	0.00
30–39	5	4	1	10	2	B-1	1.90	0.38
40–49	17	11	6	34	9	B-4; U-1	8.57	0.50
50–59	8	4	4	16	2	B-1	1.90	0.24
60–69	14	10	4	28	6	B-3	5.71	0.41
70–79	38	20	18	76	17	B-8; U-1	16.19	0.43
80–89	65	30	35	130	41	B-17; U-7	39.05	0.60
90–99	45	17	28	90	28	B-11; U-6	26.67	0.59
100–109	3	0	3	6	0	0	0.00	0.00

Sum	200	99	101	400	105	B-45; U-15	100.00	

^*^CT-studies of both knees.

^**^B: bilateral; U: unilateral.
